# Transplantation of neural stem cells in the mouse model of ischemic brain stroke and expression of genes involved in programmed cell death

**DOI:** 10.3325/cmj.2018.59.203

**Published:** 2018-10

**Authors:** Valentina Hribljan, Iva Salamon, Arijana Đemaili, Ivan Alić, Dinko Mitrečić

**Affiliations:** 1 Croatian Institute for Brain Research, University of Zagreb School of Medicine, Zagreb, Croatia; 2 Lee Kong Chian School of Medicine, Nanyang Technological University, Singapore, Singapore

## Abstract

**Aim:**

To analyze how neural stem cells (NSC) transplantation in the stroke-affected mouse brain influences the expression of genes involved in apoptosis-inducing factor (AIF)-mediated cell death – apoptosis inducing factor mitochondria associated 1 (*Aifm1*), ring finger protein 146 (*Rnf146, Iduna*), and cyclophilin A (*CypA*); necroptosis –receptor interaction protein kinase 1 (*Ripk1*), *Ripk3*, and mixed-lineage kinase domain-like protein (*Mlkl*); and apoptosis – Caspase 3 (*Casp3*) and *Casp8*.

**Methods:**

Four groups of animals were used to obtain mRNA for quantitative reverse transcription polymerase chain reaction analysis: healthy animals (n = 3), animals with stroke (n = 4), animals with stroke treated by stem cell transplantation (n = 7), and animals with stroke treated by proliferation-supporting medium (n = 5). Ischemic brain injury was induced by transient left middle cerebral artery occlusion. Statistical analysis was performed using one-way analysis of variance with *post-hoc* Tukey test.

**Results:**

NSC transplantation in the stroke-affected mouse brain significantly increased the expression of *Iduna* (*P* < 0.05), a gene-encoding protein with well-known protective effects on hypoxic damage, and significantly down-regulated the expression of damage-supportive genes, *Casp3 (P <* .01) and *Aifm1* (*P* < 0.001). We were able to distinguish between the effect produced by stem cell transplantation (*Iduna*, *Aifm1*, *Ripk3*, *Mlkl*) and the effect produced by supporting the tissue with proliferation-supporting medium (*Ripk1*, *Casp8*).

**Conclusion:**

Beside revealing some clearly positive effects of stem cells transplantation on the stroke-affected brain, our results suggest that the tissue response triggered by stem cells points toward the desired, regeneration-supporting levels of expression of a certain gene at a certain time point.

Ischemic brain stroke is one of the most devastating medical problems of the modern society, affecting more than 20 million people every year. One fourth of the patients die and another fourth remain permanently disabled. The only available pharmacological treatment is intravenous administration of recombinant tissue plasminogen activator, with a very limited number of candidates for its application (1). Despite advances in understanding of acute ischemic stroke mechanisms, more knowledge about the cellular pathways is needed to pave the way for the introduction of promising therapies such as stem cell transplantation.

Hypoxia/ischemia of the nervous tissue causes glutamate release and leads to subsequent oxidative toxicity and excitotoxicity, which are combined with the activation of molecular pathways leading to programmed cell death (2,3). Some proteins, such as apoptosis-inducing factor (AIF) and cyclophilin A (CypA), important for AIF translocation to the nucleus (4), increase cell death. Others are activated as a part of cell rescue reaction, such as ring finger protein 146 (RNF146, also called IDUNA), involved in DNA repair (5), which inhibits cell death after ischemia by preventing AIF translocation from the mitochondria (6). Growing evidence suggests that another modality of cell death, necroptosis, is specifically important in brain ischemia. Necroptosis is a type of regulated necrosis (7) involved in the pathophysiology of ischemic stroke (8). It is mediated by receptor interaction protein kinase 1 (RIPK1), RIPK3, and mixed-lineage kinase domain-like protein (MLKL). When activated by death receptors, RIPK1 and RIPK3 interact in a complex called necroptosome and phosphorylate MLKL, which then oligomerizes and leads to necroptotic plasma membrane permeabilization (9). RIPK1 inhibition by necrostatin-1 (10) and MLKL inhibition by necrosulfonamide protect against ischemic brain injury (8,11). On the other hand, active Caspase 8 (CASP8) inhibits necroptosis by cleaving RIPK1 and RIPK3, thus directing the cell to other types of responses (12).

There is growing evidence that stem cells transplantation measurably reduces the stroke area and accelerates recovery, both in preclinical (13-15) and clinical (16,17) trials. However, since the transplanted stem cells’ mechanisms of action in the injured tissue are not yet fully understood, we analyzed the influence of stem cell transplantation on the expression of genes involved in AIF-mediated cell death (*Aifm1*, *Iduna*, *CypA*), necroptosis (*Ripk1*, *Ripk3*, *Mlkl*), and apoptosis (*Casp3*, *Casp8*). Our hypothesis was that molecular pathways linked to cell death were involved in the ischemic stroke pathophysiology and that stem cells transplantation influenced their expression.

## MATERIALS AND METHODS

This study was performed in the Laboratory for Stem Cells, Croatian Institute for Brain Research, University of Zagreb School of Medicine in the period 2016–2018. All experiments were carried out in accordance with the EU Directive 2010/63/EU on the protection of animals used for scientific purposes and were approved by the Ministry of Agriculture (322-01/16-01/6) on September 1, 2015 and the Ethics Committee of the University of Zagreb School of Medicine (04-77/2010-238) on March 12, 2015.

### Experimental design

Nineteen experimental animals were divided in 4 groups as follows: age-matched healthy mice (n = 3), stroke-affected mice (n = 4), stroke-affected mice transplanted with neural stem cells (NSC) (n = 4 for *Iduna*, *CypA*, and *Casp8*, n = 5 for *Ripk1*, *Ripk3*, *Mlkl*, and *Casp3*, and n = 7 for *Aifm1*), and stroke-affected mice injected with growth factor-enriched medium without cells (n = 4 for *Iduna*, *CypA*, and *Casp8*, n = 5 for *Aifm1*, *Ripk1*, *Ripk3*, *Mlkl*, and *Casp3*). Stereotaxic injection of NSC or medium was performed 24 hours after brain injury. All procedures were performed by the same operator (IA), and animals were monitored every day. Two weeks after the stereotaxic injection, mice were sacrificed and tissues were harvested. We used animals with well-visible cortico-striatal stroke. After decapitation, the olfactory bulbs and cerebellum were removed, brains were divided into the left and right hemisphere, snap frozen in liquid nitrogen, and stored at -80°C. Total RNA was isolated, and quantitative reverse transcription polymerase chain reaction (qRT-PCR) analysis was performed.

### Animals and housing

Two mice strains were used: C57Bl/6NCrl (males for middle cerebral artery occlusion) and B6.Cg-Tg(Thy1-YFP)16Jrs/J (females for NSC isolation, The Jackson Laboratory, Bar Harbor, ME, USA). The animals were kept in the animal facility at the Croatian Institute for Brain Research at the temperature 22 ± 2°C, with 55% ± 10% humidity, and 12/12 h light/dark cycle. Water and pelleted food were given *ad libitum*. Genotyping for strain confirmation for Thy1-YFP strain was performed using the published protocols (18).

### Neural stem cells and cell culture

NSC were isolated from the telencephalic wall of the E14.5 fetuses as described previously (19). Briefly, on the day 14.5 of pregnancy, females were sacrificed, and NSC were isolated using Accutase (Gibco by Life Technologies, A11105-01, Grand Island, NY, USA). Isolated cells were placed into the flasks in a specific proliferation-supporting medium comprising DMEM/F-12 (Gibco by Life Technologies, 31331-028), B-27 Supplement (Gibco by Life Technologies, 17504-044), N-2 Supplement (Gibco by Life Technologies, 17502-048), Penicillin Streptomycin (Gibco by Life Technologies, 15070-063), FGFb (Recombinant Mouse Fibroblast Growth Factor-basic, PMG0035), and EGF (Recombinant Mouse Epidermal Growth Factor, PMG8041). Cells were cultivated in suspension, and after two days neurospheres were formed. Neurospheres were dissociated by Accutase and re-suspended in the concentration of 1000 000 cells in 1 µL of proliferation-supporting medium.

### Mouse stroke model – transient middle cerebral artery occlusion

Ischemic brain injury was induced by transient left middle cerebral artery occlusion (tMCAO) (20) in 3-month-old wild type mice weighing 25-30 g. Surgery was performed under inhalation anesthesia, mixture 2% isoflurane in 100% O_2_, under dissection microscope (Stemi DV4 Spot, Zeiss, Oberkochen, Germany). During surgery, animal body temperature was maintained with a heating pad. Following ventral neck surgery and blood vessels preparation, intraluminal filament (Doccol Company, Sharon, MA, USA) was inserted through the common carotid artery into the internal carotid artery, rostral to the origin of the middle cerebral artery, and left for 90 minutes. After 90 minutes, intraluminal filament was withdrawn, perfusion was restored, and the animal intraperitoneally received analgetic buprenorphine (0.03 mg/kg).

### Stereotaxic injection into the mouse brain

For stereotaxic injection, we used stroke-affected mice that received NSC and stroke-affected mice that received proliferation-supporting medium alone. Stroke-affected mice were injected 24 hours after the brain injury. Cells or proliferation-supporting medium were injected into the striatum (Anterior-Posterior −0.5, Medial-Lateral +2.5 and Dorsal-Ventral −2.5) according to the stereotaxic atlas using KOPF stereotaxic apparatus 900LS (David Kopf Instruments, Tujunga, CA, USA) and Hamilton syringe needle 1 µL. For anesthesia, Avertin (Sigma-Aldrich, St. Louis, MO, USA; T48402-5G) was used at a dose of 0.5 g/kg, given intraperitoneally. After injection, mice were placed on a warm heating pad, allowed to wake up, and returned to their cages.

### Tissue harvesting and storage

Two weeks after stroke, mice were sacrificed by cervical dislocation, and each hemisphere was snap-frozen in liquid nitrogen and stored at -80°C.

### Quantitative reverse transcription polymerase chain reaction

Total RNA was isolated by commercial RNeasy® Mini Kit (Qiagen, Hilden, Germany) following the manufacturer’s instructions. Concentration was calculated on a spectrophotometer (Nanodrop, Thermo Fisher Scientific, Waltham, MA, USA), and RNA was converted to single-stranded cDNA using high capacity RNA-to-cDNA Kit (Applied Biosystems, Foster City, CA, USA). Assays used in this study are listed in Table 1, while the normalization was performed with β-Actin (ACTB MGB 4352933E) (TaqMan Gene Expression Assays; Thermo Fisher Scientific). All samples were made in duplicate with 1 µg cDNA in a total volume of 20 µL using Applied Biosystems 7500 Real-Time PCR System. The comparative CT method (2^-ΔCT^) was used for relative gene expression analysis.

**Table 1:** TaqMan® Gene Expression Assays (Applied Biosystems, Foster City, CA, USA) used in this study

Assay	Identifier
*Aifm1*	Mm00442548_m1
*Rnf146 (Iduna)*	Mm00509629_m1
*CypA*	Mm02342430_g1
*Ripk1*	Mm00436354_m1
*Ripk3*	Mm00444947_m1
*Mlkl*	Mm01244222_m1
*Casp3*	Mm01195085_m1
*Casp8*	Mm01255716_m1

### Statistical analysis

The sample number was determined by availability of experimental animals and it corresponds to standards in such experiments (21). The normality of distribution was tested using Shapiro-Wilk test. All variables are expressed as mean ± standard deviation. Statistical significance of between-group differences was tested using the analysis of variance (ANOVA) with *post-hoc* Tukey test. The level of significance was set at *P* < 0.05, *P* < 0.01, and *P* < 0.001. Statistical analysis was performed using GraphPad Prism 5 (La Jolla, CA, USA, free trial version registered to our Laboratory).

## RESULTS

### Expression of genes involved in AIF-mediated cell death

**Figure 1:**
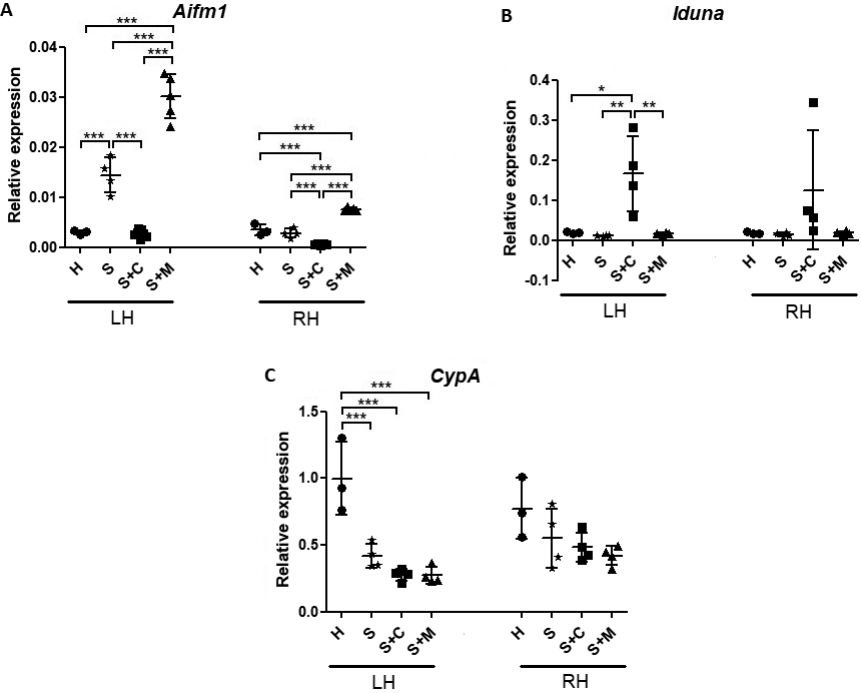
Relative expression levels of apoptosis-inducing factor mitochondria associated 1 (*Aifm1*) (**A**), ring finger protein 146 (*Rnf146, Iduna*) (**B**), and cyclophilin A (*CypA*) (**C**) in the ipsilateral (left, LH) and contralateral (right, RH) hemisphere. Groups involved include healthy mice (circles, H), stroke-affected mice (stars, S), stroke-affected mice transplanted with neural stem cells (squares, S+C), and stroke-affected mice transplanted with proliferation-supporting medium without cells (triangles, S+M). mRNA levels of each gene are shown as a percentage of endogenous control levels (*β-Actin*). Whiskers show mean ± standard deviation. Differences between groups were considered significant if *P* < 0.05 (*), *P* < 0.01(**), and *P* < 0.001 (***).

*Aifm1* level in the ipsilateral hemisphere was significantly increased by stroke (*P* < 0.001) (Figure 1A) and significantly reduced by NSC transplantation (*P* < 0.001). Injection of proliferation-supporting medium, surprisingly, increased its level in both hemispheres (Figure 1A). In the contralateral hemisphere, *Aifm1* level was not increased by stroke, but was significantly reduced by NSC transplantation (*P* < 0.001), when compared to both healthy and stroke-affected group.

*Iduna* level in the ipsilateral hemisphere was down-regulated by stroke, but not significantly (Figure 1B). It was significantly increased by NSC transplantation (*P* < 0.01) and non-significantly increased by the injection of proliferation-supporting medium. The same pattern was observed in the contralateral hemisphere, but without significant differences (Figure 1B).

*CypA* level in the ipsilateral hemisphere was significantly decreased by stroke (Figure 1C), NSC transplantation, and the injection of proliferation-supporting medium (*P* < 0.001, for all groups). In the contralateral hemisphere, a similar pattern was observed, but without significant differences (Figure 1C).

### Expression of genes involved in necroptosis and apoptosis

**Figure 2:**
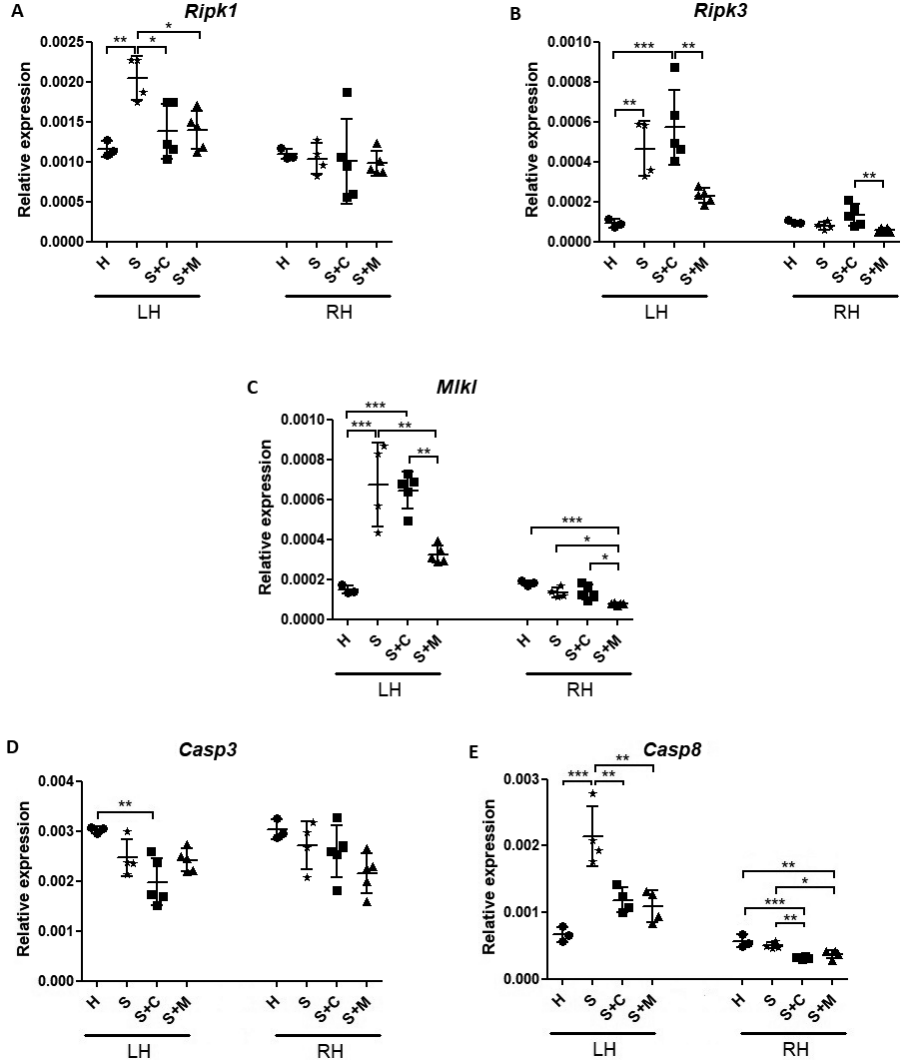
Relative expression levels of receptor interaction protein kinase 1 (*Ripk1*) (**A**), *Ripk3* (**B**), mixed-lineage kinase domain-like protein (*Mlkl*) (**C**), caspase 3 (*Casp3*) (**D**) and *Casp8* (**E**) in the ipsilateral (left, LH) and contralateral (right, RH) hemisphere. The following groups were involved: healthy mice (circles, H), stroke-affected mice (stars, S), stroke-affected mice transplanted with neural stem cells (squares, S+C), and stroke-affected mice transplanted with proliferation-supporting medium without cells (triangles, S+M). mRNA levels of each gene are shown as the percentage of endogenous control levels (*β-Actin*). Whiskers show mean ± standard deviation. Differences between groups were considered significant if *P* < 0.05 (*), *P* < 0.01(**), and *P* < 0.001 (***).

*Ripk1* and *Casp8* expression in the ipsilateral hemisphere followed the same pattern: they were significantly up-regulated by stroke (*P* < 0.01 for *Ripk1* and *P* < 0.001 for *Casp8*) and reduced by NSC transplantation and the injection of proliferation-supporting medium to levels similar to healthy mice (Figure 2A-E). In the contralateral hemisphere, there was no significant difference in *Ripk1* expression between the groups. *Casp8* expression was reduced by both NSC transplantation and the injection of proliferation-supporting medium when compared with healthy and stroke-affected group.

*Ripk3* and *Mlkl* in the ipsilateral hemisphere followed the same expression pattern: they were significantly increased by stroke (*P* < 0.01 for *Ripk3* and *P* < 0.001 for *Mlkl*) and reduced by the injection of proliferation-supporting medium (significant for Mlkl *P* < 0.01), but not by NSC transplantation (Figure 2B and 2C). This unique phenomenon was also found in the contralateral hemisphere.

*Casp3* level in the ipsilateral hemisphere was decreased after stroke compared with healthy controls, but the difference was not significant. It was significantly reduced by NSC transplantation compared with healthy mice. In the contralateral hemisphere, there was no significant difference between the groups (Figure 2D).

## DISCUSSION

**Figure 3:**
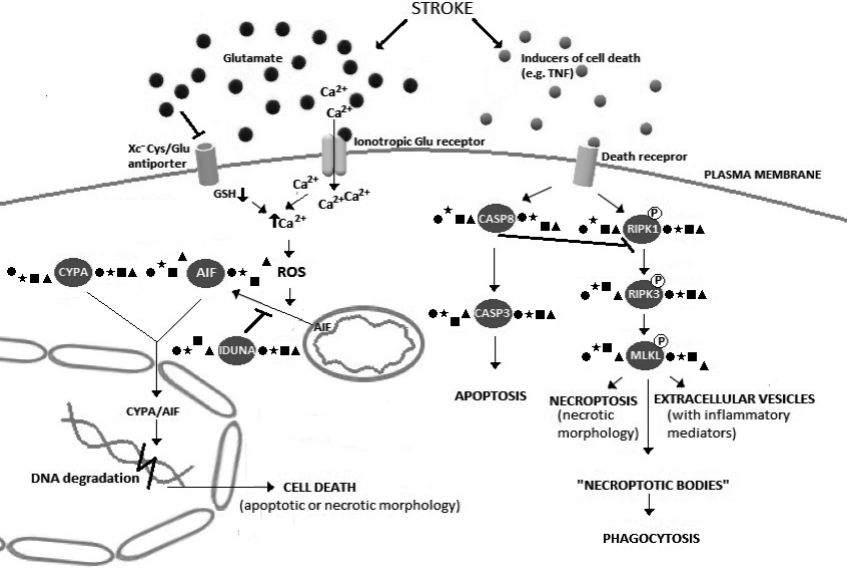
Scheme of molecular pathways of apoptosis-inducing factor (AIF)-mediated cell death, necroptosis, and apoptosis following stroke. The proteins for which genes were analyzed are shown in gray circles. Symbols on each side represent the expression pattern of genes from the corresponding hemisphere: the ipsilateral hemisphere to the left and the contralateral hemisphere to the right. Black circle represents healthy mice, star represents stroke-affected mice, square represents stroke-affected mice transplanted with neural stem cells, and triangle represents stroke-affected mice transplanted with proliferation-supporting medium without cells. Glutamate, which is released following stroke, causes oxidative stress by inhibiting cystine-glutamate antiporter (Xc-) and depleting the intracellular pool of glutathione, increasing intracellular Ca^2+^, which leads to oxidative toxicity (oxytosis) (2). Glutamate can also bind to N-methyl-D-aspartate (NMDA) receptors inducing Ca^2+^ influx and direct excitotoxicity (3). Both excitotoxicity and oxytosis cause the AIF release from outer mitochondria membrane and its translocation to the nucleus, which leads to cell death. Protein IDUNA has a protective role because it decreases AIF nuclear translocation (3). On the other hand, CYPA is a pro-cell death protein, shown to be important for AIF translocation to the nucleus, and inhibition of AIF/CYPA complex has been shown to be protective against oxidative stress-induced cell death pathways. Cell death in nervous system can also be triggered by binding of inducers of cell death to death receptors, causing at least two different types of cell death: apoptosis and necroptosis. Activated death receptors induce RIPK1 phosphorylation, which then phosphorylates RIPK3 and makes with it a complex, called necroptosome (9). The necroptosome phosphorylates mixed-lineage kinase domain-like protein (MLKL), which then oligomerizes and causes necroptotic plasma membrane permeabilization. If active Caspase 8 (CASP8) is present in the cytoplasm, it inhibits necroptosis by cleaving RIPK1 and RIPK3, and this directs the cell to apoptosis, which is executed by CASP3 (12). Besides being involved in necroptosis, MLKL is also found in the “necroptotic bodies,” which can be phagocytosed (22) and is important for the assembly of extracellular vesicles with inflammatory mediators (23). Abbreviations: *Aifm1 –*apoptosis inducing factor mitochondria associated 1; *Rnf146 –* ring finger protein 146 (*Iduna*); *CypA –* cyclophilin A; TNF *–* tumor necrosis factor; Xc^-^ Cys/Glu *–* cystine/glutamate antiporter; Ca^2+^ *–* calcium ion; GSH *–* glutathione; ROS *–* reactive oxygen species; P *–* phosphorylated; Casp *–* caspase.

We confirmed our hypothesis by showing that NSC transplantation significantly influenced the expression of genes linked to different modalities of cell death after stroke (Figure 3). Fourteen days after the onset of the ischemic brain damage, the majority of genes involved in promoting cell death exhibited increased expression levels (*Casp8*, *Aifm1*, *Ripk1*, *Ripk3*, and *Mlkl*), while one gene that opposed this direction was down-regulated (*Iduna*). In only one case, a cell death-promoting gene, *CypA* was down-regulated, which suggested the presence of a strong endogenous reparative mechanism. In addition, we showed that all three major markers of necroptosis (*Ripk1*, *Ripk3*, *Mlkl*) were significantly increased after ischemic brain damage. This corresponds to their hypothesized role in cell death in the nervous tissue following hypoxia (24).

We analyzed the potential role of transplanted NSC in supporting tissue recovery. In the majority of brain diseases, expected benefits from cell transplantation cover a whole spectrum, involving modulation of inflammation, cell rescue, and cell replacement. We have already shown that different stem cell populations exhibit different beneficial effects on disease-affected nervous tissue (25). Although some studies, both on preclinical and clinical levels, described mechanisms responsible for beneficial effects, including effects on transplanted cells (26), many of them focused on description of cell fates (27) or reported beneficial effects, while the mechanisms themselves remained elusive. Most studies analyzing cell death reported only one or a few markers, mostly focusing on classic apoptotic pathways, like *CASP3* (28). One of the rare studies that analyzed a wider array of genes in the rat model of stroke showed that transplantation of human umbilical cord mesenchymal stem cells reduced apoptosis markers (29).

To obtain insight into the possible role of the programmed cell death mediated by AIF, we analyzed the expression of *Aifm1*, *Iduna,* and *CypA*. AIF is linked to glutamate toxicity and *Aifm1* seems to be one of the most potent genes for general control over cell death. *Aifm1* levels can be reduced, leading to abnormal cell survival, as observed in some human tumor types (30), but also increased, like in the case of hypoxic damage. Our finding that *Aifm1* expression was increased after tMCAO but significantly reduced and normalized after NSC transplantation highly suggests a protective role of NSC transplantation. More importantly, we discovered positive effects of NSC transplantation, including a huge up-regulation of *Iduna* levels. IDUNA has protective effects on cells, including its strong antioxidative (31) and antiglutamate (32) effects and effects linked to the rescue of cells from a fatal G1 arrest and facilitation of DNA repair (5). Specific for brain damage, *Iduna* was clearly shown to be down-regulated in brain ischemia, which is directly linked to increased cell damage (32). Moreover, the rescue of *Iduna* in neurons correlates with ischemic damage decrease (33). Our study is the very first to report that NSC transplantation significantly up-regulates *Iduna* levels. This means that NSC simultaneously down-regulate *Aifm1* and up-regulate *Iduna*, which is a combination with a possibly enormous protective effect on ischemia-affected tissue. Furthermore, we also showed that NSC transplantation after a stroke caused one more positive effect – *Casp3* down-regulation.

By selecting 14 days after stroke onset as the measurement time point, we deliberately aimed at the transition from sub-acute to chronic phase, including both removal of cells damaged by ischemia and regeneration. This raises the question of whether the significantly down-regulated or up-regulated gene levels are linked to the pathological process occurring, thus mirroring the negative events, or completely opposite – whether the remaining tissue aims to change the gene expression levels because this is needed for regeneration. Our study suggests that the part of the answer can be found in the tissue treated by transplanted stem cells. The presence of transplanted stem cells supports regenerative events (13,14,17). For example, stroke increases the levels of *Ripk1*, a necroptosis promotor, while transplanted medium and NSC oppose this process (Figure 2A). The opposite example is *CypA*. Its down-regulation 14 days after stroke onset cannot be observed in the same way as *Iduna* down-regulation. *Iduna* is a well-known protective gene, whose down-regulation is a marker of negative effect of hypoxia, corrected by up-regulation found after NSC transplantation. Indeed, in this case, *CypA* down-regulation after 14 days is likely an endogenous regenerative, protective response of the brain tissue, as was seen in animals treated with NSC in our study. Bearing this in mind, the pattern observed for *Ripk1*, *Ripk3,* and *Mlkl* is most interesting. While both the proliferation-supporting medium and NSC significantly reduced *Ripk1* expression, they had different effects on *Ripk3* and *Mlkl*: the medium suppressed *Ripk3* and *Mlkl* expression and NSC increased it. RIPK1 inhibition by necrostatin-1 (10) and MLKL inhibition by necrosulfonamide protects against ischemic brain injury (8,11). On the other hand, RIPK3-deficient mice were not protected against tMCAO-induced stroke compared with their wild type counterparts (34). This leads to the suggestion that NSC transplantation yields an opposite effect, that is, that it maintains the high *Ripk3* and *Mlkl* levels. One of the possible explanations could be that *Ripk1* is the major genetic activator of the necroptotic complex. Its controlling action is very clearly negative for the ischemia-affected tissue. Moreover, MLKL regulates endosomal trafficking, while RIPK3 boosts vesicles release, which altogether withholds cell death (23). Our results, therefore, suggest that, when aiming at necroptosis, *Ripk1* should be decreased, while *Ripk3* and *Mlkl* activation might not be so harmful.

This study analyzed gene expression in the whole hemisphere, which presents a limitation because we cannot determine in which cell type the observed changes occur. For example, we showed that *Casp8*, *Ripk1,* and *Ripk3* were up-regulated following stroke, although it has been shown that CASP8 blocks assembly of necroptotic complex composed of RIPK1 and RIPK3, leading the cell to apoptosis (12). It is possible that *Casp8* might be up-regulated in one cell type and *Ripk1* and *Ripk3* in another. Also, we showed that *Ripk3* and *Mlkl* levels were up-regulated following stroke and reduced by the proliferation-supporting medium, but their high expression levels were maintained by NCS. MLKL, besides being involved in necroptosis, is found in the “necroptotic bodies,” which can be phagocytosed (22), and is involved in assembling of extracellular vesicles, which contain inflammatory mediators (23). Therefore, *Ripk3* and *Mlkl* up-regulation might not be as harmful as previously thought, and in some cases might be needed for tissue recovery. Further research is needed to determine in which cell type and brain region this up-regulation occurs and whether its role is protective or damage-supportive. In addition, a limitation of our study was that the sample size was determined according to previous similar research and was not calculated before conducting the research. However, *post-hoc* power analysis with test strength of 80% confirmed that the number of animals per group was sufficient.

In conclusion, this study revealed an important role of different types of cell death in the pathophysiology of ischemic stroke. Even more importantly, it demonstrated that NSC transplantation up-regulated *Iduna*, a gene linked to regeneration, and down-regulated genes linked to cell death (*Aifm1*, *Casp3*). Moreover, our model can be used to get insight in the effects of a certain gene at a certain time point. Direction of change in gene expression after NSC transplantation, especially if this change is very pronounced and significant, may suggests whether it is beneficial to activate or to block the expression of a certain gene if regeneration of hypoxia-affected tissue is to be improved.
